# KL-6 levels in COVID-19

**DOI:** 10.37825/2239-9747.1014

**Published:** 2020-10-01

**Authors:** G Scarpati, B Polverino, M Boffardi, O Piazza

**Affiliations:** 1 University of Salerno; 2 AOU San Giovanni di Dio e Ruggi d’Aragona

**Keywords:** KL-6, COVID-19, ARDS

Dear Editor,

the Krebs von den Lungen 6 (KL-6) is a high molecular weight mucin-like glycoprotein produced by type II pneumocytes and bronchial epithelial cells. In the acute respiratory distress syndrome (ARDS) disruption of the alveolar epithelium is described. Since elevated circulating levels of KL-6 are thought to indicate disruption of the alveolar epithelial lining, we assumed it could be advantageous to analyzing KL-6 in a very severe group of COVID-19 positive patients, with a PaO_2_/FiO_2_ ratio ≤100, admitted in ICU for ARDS, who underwent mechanical ventilation.

In a previously letter, Frix et al [1] retrospectively compared KL-6 serum levels among a cohort of 83 COVID-19 patients, and versus two other groups, one of healthy subjects (n = 70) and the other suffering from interstitial lung diseases (n = 31). In the above-mentioned paper, higher levels of KL-6 in COVID-19 patients were associated with worse oxygen levels at admission (in the ambient air, median SpO_2_ 90% in high KL-6 level patients vs 94% in low KL-6 level patients; p = 0.013). However, high KL-6 were not linked to severe dyspnea (p = 0.585), or to ICU admission (p = 0.434). In 5 consecutive ICU patients, we found median KL-6 serum value higher than the value reported by Frix et al. On the ICU admission, in fact, it ranged from 163 U/mL to 3126 U/mL, with median value 757,5 U/mL (IQ 581–1853), while in Frix et al median value was 405 (IQ 277–592). This difference may be explained by the greater severity of the Salerno University Hospital patients. In fact, D’Alessandro et al [2], in a study including 22 patients, of which 12 underwent intubation and mechanical ventilation in the COVID ICU (severe group), reported results similar to our pilot data: KL-6 serum concentrations were significantly higher in severe patients (1021U/mL; IQ 473–1909) than the less severe group (293 [197–362]; p = .0118).

Although our outcomes are absolutely preliminary, if taken together with other scientific groups results, they encourage KL-6 evaluation as a potential biomarker for phenotyping patients according to disease severity. It would be very important to establish its prognostic potential too. Nevertheless, the search for credible biomarkers is still open, and proteomic data, together with artificial intelligence, i.e., machine learning, can probably expand our findings [3].

## Abbreviations

KL-6: Krebs von den Lungen 6
ARDS: acute respiratory distress syndrome
ICU: Intensive Care Unit

## References

[1] Frix A, Schoneveld L, Ladang A (2020). Could KL-6 levels in COVID-19 help to predict lung disease?. Respir Res.

[2] D’Alessandro M, Cameli P, Refini RM (2020). Serum KL-6 concentrations as a novel biomarker of severe COVID-19. J Med Virol.

[3] Shu T, Ning W, Wu D (2020). Plasma Proteomics Identify Biomarkers and Pathogenesis of COVID-19. Immunity.

